# Kinetic data analysis of chaperone-like activity of Wt, R69C and D109H αB-crystallins

**DOI:** 10.1016/j.dib.2019.104922

**Published:** 2019-12-04

**Authors:** Maryam Ghahramani, Reza Yousefi, Alexey Krivandin, Konstantin Muranov, Boris Kurganov, Ali Akbar Moosavi-Movahedi

**Affiliations:** a Protein Chemistry Laboratory (PCL), Department of Biology, College of Sciences, Shiraz University, Shiraz, Iran; b Emanuel Institute of Biochemical Physics, Russian Academy of Sciences, Kosygin str. 4, Moscow 119991, Russia; c Bach Institute of Biochemistry, Research Center of Biotechnology of the Russian Academy of Sciences, 33, bld. 2 Leninsky Ave., Moscow 119071, Russia; d Institute of Biochemistry and Biophysics (IBB), University of Tehran, Tehran, Iran

**Keywords:** Human αB-crystallin, Chaperone-like activity, Kinetic data, Aggregation, Light scattering

## Abstract

The α-Crystallin (α-Cry) functions as a molecular chaperone, preventing the formation of stress-induced protein aggregation which is important for maintenance of lens transparency. The kinetic data of Wt, R69C and D109H αB-Crys chaperone-like activity were obtained by UV–Vis spectroscopy in both thermal- and chemical-induced aggregation methods. The data were analyzed using physical parameters describing the aggregation process including t* (the characteristic of the stage of nucleation), and *t*_0.5_ (the characteristic of the stage of aggregate growth) and *I*_lim_ (the limiting value of the light scattering intensity). Parameter t* is duration of the lag phase and the lower t* value is associated with the higher rate of the nucleation stage. Also, the lower values of *t*_0.5_ indicated the higher rate of aggregate growth stage. The change in parameter *I*_lim_ in the presence of chaperones can be connected with the change in the size of protein aggregates. These data are related to the research article entitled “*Structural and functional characterization of D109H and R69C mutant versions of human αB-crystallin: the biochemical pathomechanism underlying cataract and myopathy development*” [1].

**Table:** Specifications Table

Subject	Biochemistry
Specific subject area	αB-crystallin, Chaperone-like activity, Aggregation
Type of data	Graphs and tables of kinetic data analyses
How data were acquired	Protein aggregation assessment by monitoring light scattering at 360 nm as a function of time, using a T90^+^ UV–Vis spectrophotometer (PG Instrument Ltd., UK) equipped with a Peltier temperature controller.
Data format	Raw and analyzed
Parameters for data collection	Chaperone-like activity of Wt, R69C and D109H αB-Crys was evaluated with different client proteins including: insulin, lysozyme, catalase and γ-Cry, in both thermal- and chemical-induced aggregation methods.
Description of data collection	Aggregation of different client proteins in the absence and presence of chaperones was assessed by monitoring light scattering at 360 nm as a function of time, using UV–Vis spectroscopy.
Data source location	Shiraz University, Shiraz, Iran
Data accessibility	With the article
Related research article	M. Ghahramani, R. Yousefi, A. Krivandin, K. Muranov, B. Kurganov, A.A. Moosavi-Movahedi, Structural and functional characterization of D109H and R69C mutant versions of human αB-crystallin: the biochemical pathomechanism underlying cataract and myopathy development, Int. J. Biol. Macromol. S0141-8130 (2019) 34809-3. doi: 10.1016/j.ijbiomac.2019.09.239.

**Table:** 

**Value of the Data** • The data provide a further mechanistic insight into anti-aggregation ability of human αB-Cry and its mutant forms (R69C and D109H). • The data might be used for modulating chaperone activity of the mutant proteins using chemical chaperones. • These data also show the effect of each chaperone on the important parameters shaping chaperoning activity. • These data clearly display the client protein-specific chaperone activity of the mutant proteins.

## 1 Data

### 1.1 Kinetic data analysis of chaperone-like activity of different αB-Crys

The aggregation process, obeying the mechanism of nucleation-dependent aggregation, involves the stage of nucleation and the stage of aggregate growth. When studying the aggregation kinetics by registration of increment of the light scattering intensity, the following equation is often applicable for description of the dependence of the light scattering intensity on time [[2], [3], [4]]:

(1) $$I=I_{\lim}\left\{1-\exp\left[-k_{\text{I}}\left(t-t*\right)\right]\right\},\left(t\text{ > }t\text{∗}\right)$$

where *k*_I_ is the rate constant of the first order, *I*, *I*_0_ and *I*_lim_ are the current, initial (at *t* = 0) and limiting (at *t* → ∞) values of the light scattering intensity and t* is a point in time corresponding to crossing of the theoretical curve, which calculated with this equation, with the horizontal line *I* = 0 calculated with this equation. Parameter t* is duration of the lag phase and may be considered as a characteristic of the rate of the nucleation stage. The lower the t* value, the higher is the rate of the nucleation stage. Eq. (1) can be transformed as follows:

(2) $$I=I_{\lim}\left\{1-\exp\left[-\left(\ln\;2\right)\left(t-t*\right)/t_{0.5}\right]\right\}$$

(*t*_0.5_ = ln2/*k*_I_)

The physical sense of parameter *t*_0.5_ is the following. At *t* = (t* + *t*_0.5_) the value of *I* is equal to *I*_lim_/2. Parameter *t*_0.5_ may be considered as a characteristic of the rate of the stage of aggregate growth. The lower the *t*_0.5_ value, the higher is the rate of the stage of aggregate growth. The change in parameter *I*_lim_ in the presence of chaperones can be connected with the change in the size of protein aggregates. The diminishing of the *I*_lim_ value in the presence of chaperones can be due to the decrease in the size of protein aggregates.

#### 1.1.1 Aggregation of insulin in the presence of 20 mM DTT (42 °C)

**Fig. 1:**
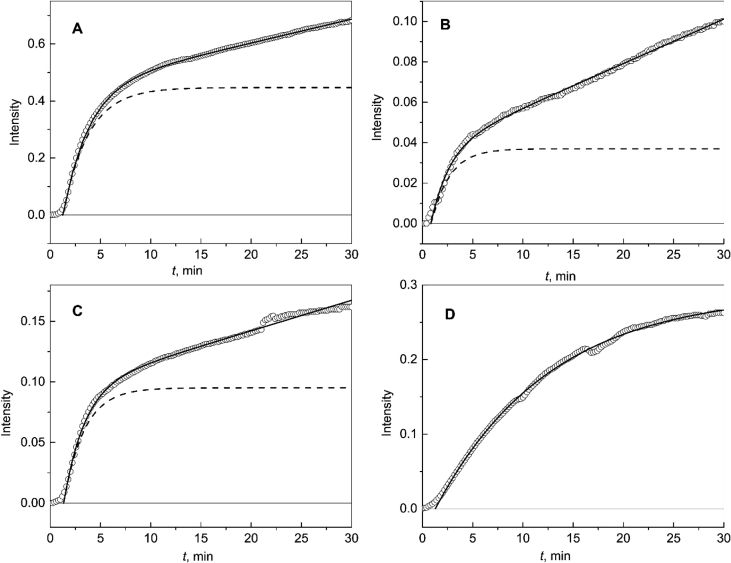
**Aggregation of insulin (0.3 mg mL**^**−1**^**) in the presence of 20 mM DTT at 42 °C.** (A) The dependence of the light scattering intensity (*I*) on time (*t*) for aggregation of insulin in the absence of any additives. Points are experimental data. Solid curve was calculated from Eq. (3) at the following values of parameters: *I*_lim_ = 0.447, t* = 1.25 min, *t*_0.5_ = 1.76 min and *B* = 0.00834 min^−1^. Dotted curve was calculated from Eq. (2) at the following values of parameters: *I*_lim_ = 0.447, t* = 1.25 min and *t*_0.5_ = 1.76 min. (B) The dependence of *I* on *t* for aggregation of insulin in the presence of Wt αB-Cry (0.08 mg mL^−1^). Solid curve was calculated from Eq. (3) at the following values of parameters: *I*_lim_ = 0.037, t* = 0.84 min, *t*_0.5_ = 1.26 min and *B* = 0.00220 min^−1^. Dotted curve was calculated from Eq. (2) at the following values of parameters: *I*_lim_ = 0.037, t* = 0.84 min and *t*_0.5_ = 1.26 min. (C) The dependence of *I* on *t* for aggregation of insulin in the presence of R69C mutant form of αB-Cry (0.08 mg mL^−1^). Solid curve was calculated from Eq. (3) at the following values of parameters: *I*_lim_ = 0.095, t* = 1.33 min, *t*_0.5_ = 1.42 min and *B* = 0.00252 min^−1^. Dotted curve was calculated from Eq. (2) at the following values of parameters: *I*_lim_ = 0.095, t* = 1.33 min and *t*_0.5_ = 1.42 min. (D) The dependence of *I* on *t* for aggregation of insulin in the presence of D109H mutant form of αB-Cry (0.08 mg mL^−1^). Solid curve was calculated from Eq. (2) at the following values of parameters: *I*_lim_ = 0.289, t* = 1.28 min, *t*_0.5_ = 7.87 min.

Fig. 1A shows the kinetics of DTT-induced aggregation of insulin at 42 °C. The initial kinetic data are represented in Table S1 in supplementary materials [1]. As can be seen from this Figure, at rather high values of time the light scattering intensity increases linearly with increasing time. Taking into account this peculiarity of the shape of the kinetic curve, the following equation can be proposed for description of the dependence of the light scattering intensity on time:

(3) $$I=I_{\lim}\left\{1-\exp\left[-\left(\ln\;2\right)\left(t-t*\right)/t_{0.5}\right]\right\}+B\left(t-t*\right),$$

where *B* is constant. This equation was used to describe the kinetic curves of insulin aggregation in the absence of any additives (Fig. 1A, *B* = 0.00834 ± 0.00006 min^−1^) and in the presence of Wt αB-Cry (Fig. 1B; *B* = 0.00220 ± 0.00002 min^−1^) and in the presence of R69C mutant form of αB-Cry (Fig. 1C; *B* = 0.00252 ± 0.00004 min^−1^). When studying the effect of D109H mutant form of αB-Cry on insulin aggregation, Eq. (2) was used for description of the kinetic curve (*B* = 0). Parameters *I*_lim_, t* and *t*_0.5_ for insulin aggregation calculated using theoretical equations (2), (3) are given in Table 1.

**Table 1:** Analyzed parameters for kinetic data on different client-protein aggregation.

Additives	*I*_lim_	t*, min	*t*_0.5_, min	*m*	*R*^2^
DTT-induced aggregation of insulin at 42 °C					
No additives	0.447 ± 0.001	1.25 ± 0.01	1.76 ± 0.02	1	0.9994
αB-crystallin Wt	0.037 ± 0.001	0.84 ± 0.04	1.26 ± 0.05	1	0.9976
αB-crystallin R69C	0.095 ± 0.001	1.33 ± 0.04	1.42 ± 0.05	1	0.9953
αB-crystallin D109H	0.289 ± 0.001	1.28 ± 0.04	7.87 ± 0.09	1	0.9985
Aggregation of catalase at 60 °C					
No additives	1.06 ± 0.02	2.55 ± 0.02	3.42 ± 0.13	3.16 ± 0.11	0.9989
αB-crystallin Wt	0.110 ± 0.001	1.83 ± 0.02	3.65 ± 0.05	1.93 ± 0.06	0.9989
αB-crystallin R69C	0.0384 ± 0.0005	1.65 ± 0.02	1.67 ± 0.04	2.08 ± 0.10	0.9898
αB-crystallin D109H	0.600 ± 0.001	2.62 ± 0.01	3.04 ± 0.01	1.10 ± 0.01	0.9998
DTT-induced aggregation of lysozyme at 42 °C					
No additives	1.220 ± 0.007	8.34 ± 0.07	7.90 ± 0.07	1.18 ± 0.03	0.9991
αB-crystallin Wt	0.745 ± 0.003	27.7 ± 0.2	6.69 ± 0.15	0.69 ± 0.03	0.9978
αB-crystallin R69C	0.848 ± 0.001	18.0 ± 0.1	11.4 ± 0.1	0.55 ± 0.01	0.9997
αB-crystallin D109H	1.020 ± 0.007	22.2 ± 0.1	8.22 ± 0.06	1.02 ± 0.03	0.9996
Aggregation of γ-crystallin at 60 °C					
No additives	1.138 ± 0.002	9.5 ± 0.1	8.0 ± 0.1	0.75 ± 0.02	0.9987
αB-crystallin Wt	0.717 ± 0.002	20.8 ± 0.1	8.1 ± 0.1	0.69 ± 0.02	0.9988
αB-crystallin R69C	0.811 ± 0.001	21.0 ± 0.1	4.2 ± 0.1	0.85 ± 0.02	0.9978
αB-crystallin D109H	0.788 ± 0.002	15.8 ± 0.1	4.6 ± 0.1	1.03 ± 0.03	0.9976

#### 1.1.2 Aggregation of catalase at 60 °C

**Fig. 2:**
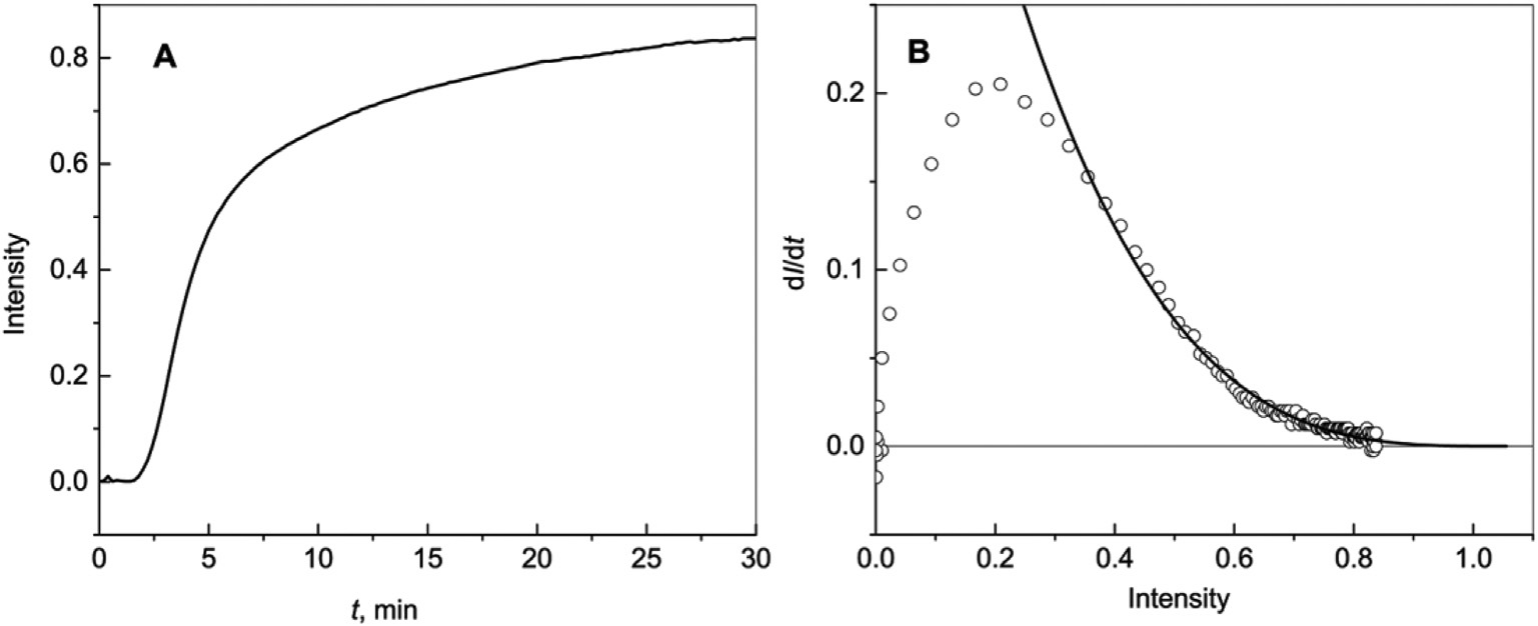
**Aggregation of catalase (0.3 mg mL**^**−1**^**) at 60 °C.** (A) The dependence of the light scattering intensity (*I*) on time (*t*). (B) The dependence of derivative d*I*/d*t* on the light scattering intensity. Points are experimental data. Solid curve was calculated from Eq. (4) at the following values of parameters: *D* = 0.50 min^−1^, *I*_lim_ = 1.06 and *m* = 3.4.

Fig. 2A shows the kinetics of aggregation of catalase at 60 °C. The initial kinetic data are represented in Table S2 in supplementary materials. To analyze the shape of the kinetic curve, we have constructed the dependence of derivative d*I*/d*t* on *I* (Fig. 2B). The dependence of d*I*/d*t* on *I* can be described by equation [3]:

(4) $$\frac{\text{d}I}{\text{d}t}=D{\left(I_{\lim}-I\right)}^{m},$$

where *D* is constant. Parameter *m* was found to be equal to 3.4 ± 0.2.

Integration of Eq. (4) gives the following expression:

(5) $$I=I_{\lim}\left\{1-\frac{1}{{\left[1+\left(2^{m-1}-1\right)\left(t-t*\right)/t_{0.5}\right]}^{1/\left(m-1\right)}}\right\}.$$

It should be noted, if *m* = 1, the dependence of the light scattering intensity on time follows Eq. (2).

**Fig. 3:**
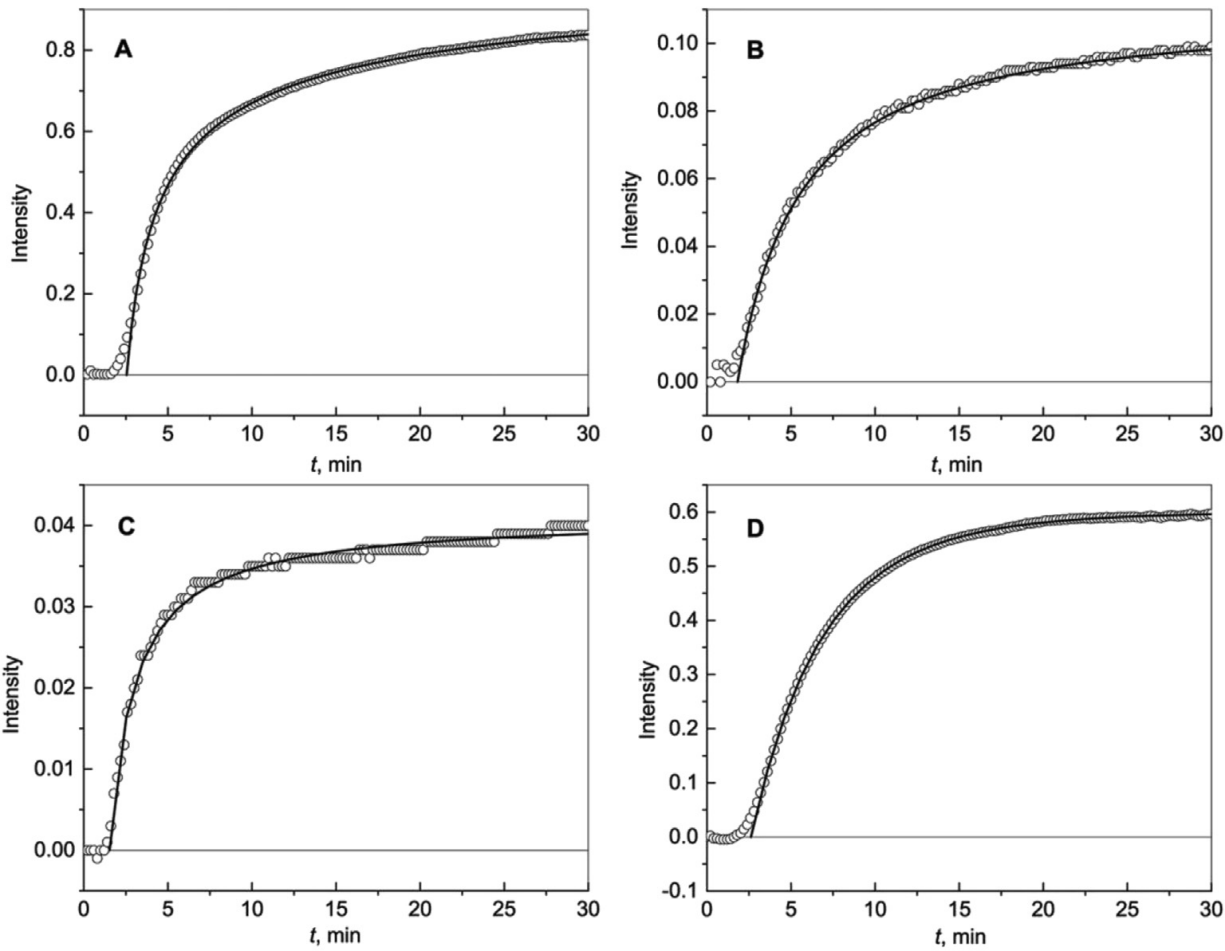
**Effect of αB-Cry and mutant forms of αB-Cry on aggregation of catalase (0.3 mg mL**^**−1**^**) at 60 °C.** (A) The dependence of the light scattering intensity (*I*) on time (*t*) for aggregation of catalase in the absence of any additives. Points are experimental data. Solid curve was calculated from Eq. (5) at the following values of parameters: *I*_lim_ = 1.06, t* = 2.55 min, *t*_0.5_ = 3.42 min and *m* = 3.2. (B) The dependence of *I* on *t* for aggregation of catalase in the presence of Wt αB-Cry (0.08 mg mL^−1^). Solid curve was calculated from Eq. (5) at the following values of parameters: *I*_lim_ = 0.110, t* = 1.83 min, *t*_0.5_ = 3.65 min and *m* = 1.93. (C) The dependence of *I* on *t* for aggregation of catalase in the presence of R69C mutant form of αB-Cry (0.08 mg mL^−1^). Solid curve was calculated from Eq. (5) at the following values of parameters: *I*_lim_ = 0.0414, t* = 1.53 min, *t*_0.5_ = 1.54 min and *m* = 2.1. (D) The dependence of *I* on *t* for aggregation of catalase in the presence of D109H mutant form of αB-Cry (0.08 mg mL^−1^). Solid curve was calculated from Eq. (5) at the following values of parameters: *I*_lim_ = 0.600, t* = 2.62 min, *t*_0.5_ = 3.04 min and *m* = 1.10.

Fig. 3 shows the kinetics of aggregation of catalase in the presence of Wt, R69C and D109H αB-Crys. Parameters *I*_lim_, t*, *t*_0.5_ and *m* calculated for the kinetic curves using Eq. (5) are given in Table 1.

#### 1.1.3 Aggregation of lysozyme in the presence of 20 mM DTT (42 °C)

**Fig. 4:**
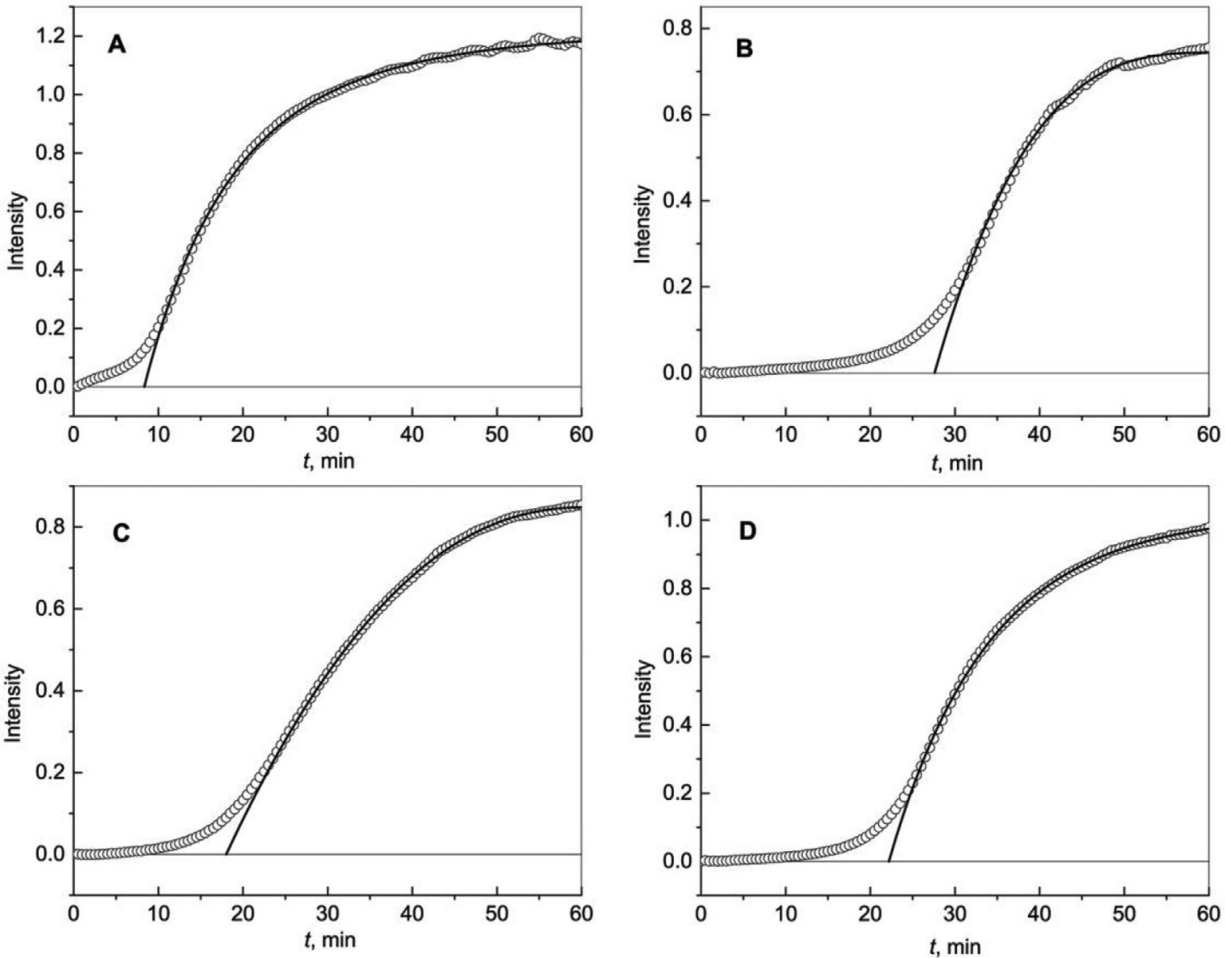
**Aggregation of lysozyme (0.2 mg mL**^**−1**^**) in the presence of 20 mM DTT at 42 °C.** (A) The dependence of the light scattering intensity (*I*) on time (*t*) for aggregation of lysozyme in the absence of any additives. Points are experimental data. Solid curve was calculated from Eq. (5) at the following values of parameters: *I*_lim_ = 1.220, t* = 8.34 min, *t*_0.5_ = 7.90 min and *m* = 1.18. (B) The dependence of *I* on *t* for aggregation of lysozyme in the presence of Wt αB-Cry (0.08 mg mL^−1^). Solid curve was calculated from Eq. (5) at the following values of parameters: *I*_lim_ = 0.745, t* = 27.7 min, *t*_0.5_ = 6.69 min and *m* = 0.69. (C) The dependence of *I* on *t* for aggregation of lysozyme in the presence of R69C mutant form of αB-Cry (0.08 mg mL^−1^). Solid curve was calculated from Eq. (5) at the following values of parameters: *I*_lim_ = 0.848, t* = 18.0 min, *t*_0.5_ = 11.4 min and *m* = 0.55. (D) The dependence of *I* on *t* for aggregation of lysozyme in the presence of D109H mutant form of αB-Cry (0.08 mg mL^−1^). Solid curve was calculated from Eq. (2) at the following values of parameters: *I*_lim_ = 1.020, t* = 22.2 min, *t*_0.5_ = 8.22 min and *m* = 1.02.

Kinetics of DTT-induced aggregation of lysozyme at 42 °C in the absence and in the presence of Wt, R69C and D109H αB-Crys (Fig. 4) was analyzed using Eq. (5). The initial kinetic data are represented in Table S3 in supplementary materials. Parameters *I*_lim_, t*, *t*_0.5_ and *m* for lysozyme aggregation are given in Table 1.

#### 1.1.4 *Aggregation of* γ-crystallin *at 60 °C*

**Fig. 5:**
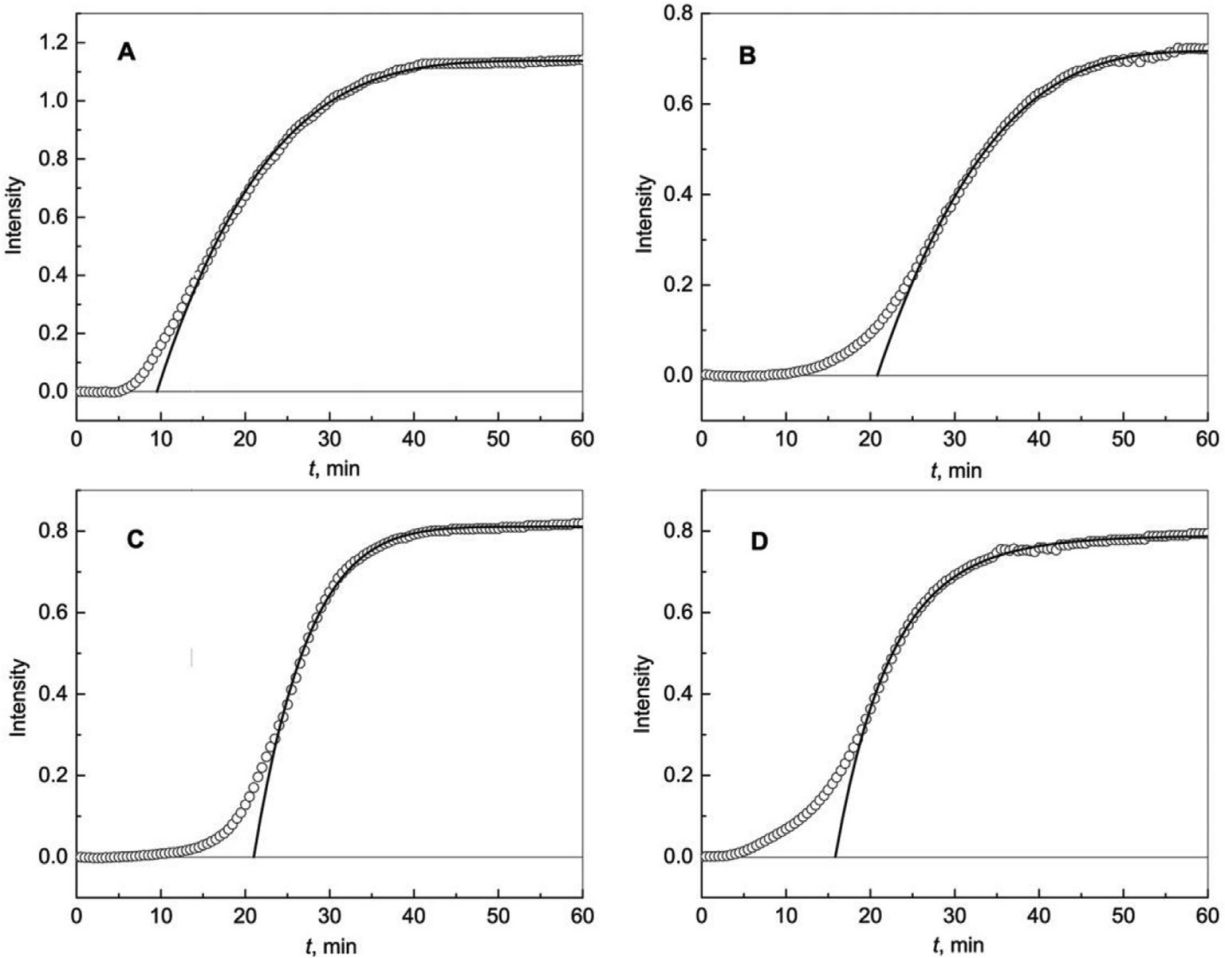
**Aggregation of γ-Cry (0.16 mg mL**^**−1**^**) at 60 °C.** (A) The dependence of the light scattering intensity (*I*) on time (*t*) for aggregation of γ-Cry in the absence of any additives. Points are experimental data. Solid curve was calculated from Eq. (5) at the following values of parameters: *I*_lim_ = 1.138, t* = 9.5 min, *t*_0.5_ = 8.0 min and *m* = 0.75. (B) The dependence of *I* on *t* for aggregation of γ-Cry in the presence of Wt αB-Cry (0.08 mg mL^−1^). Solid curve was calculated from Eq. (5) at the following values of parameters: *I*_lim_ = 0.717, t* = 20.8 min, *t*_0.5_ = 8.1 min and *m* = 0.69. (C) The dependence of *I* on *t* for aggregation of γ-Cry in the presence of R69C mutant form of αB-Cry (0.08 mg mL^−1^). Solid curve was calculated from Eq. (5) at the following values of parameters: *I*_lim_ = 0.811, t* = 21.0 min, *t*_0.5_ = 4.2 min and *m* = 0.85. (D) The dependence of *I* on *t* for aggregation of γ-Cry in the presence of D109H mutant form of αB-Cry (0.08 mg mL^−1^). Solid curve was calculated from Eq. (2) at the following values of parameters: *I*_lim_ = 0.788, t* = 15.8 min, *t*_0.5_ = 4.6 min and *m* = 1.03.

Fig. 5 shows the kinetics of aggregation of γ-crystallin (γ-Cry) at 60 °C in the absence and in the presence of Wt, R69C and D109H αB-Crys. The initial kinetic data are represented in Table S4 in supplementary materials. Parameters *I*_lim_, t*, *t*_0.5_ and *m* for lysozyme aggregation calculated using Eq. (5) are given in Table 1.

## 2 Experimental design, materials, and methods

### 2.1 Chaperone-like activity assessment of R69C and D109H mutant αB-Crys

The chaperone-like activity of mutant αB-Crys was measured using different client proteins including insulin, lysozyme, catalase and γ-Cry [5]. Aggregation of bovine pancreatic insulin (0.3 mg mL^−1^) and chicken egg white lysozyme (0.2 mg mL^−1^) was induced with dithiothreitol (DTT; 20 mM) in buffer A at 40 °C. The heat-induced aggregation of γ-Cry and bovine liver catalase was performed at 60 °C. The molar ratio of chaperone/γ-Cry was set at 1:2. The aggregation of catalase (0.3 mg mL^−1^) was induced in the presence of different chaperones. The light scattering of the client proteins was measured while the concentration of the chaperone was fixed at 0.1 mg mL^−1^. The aggregation of γ-Cry was obtained in the presence of 0.08 mg mL^−1^ of Wt and mutant αB-Cry chaperones. The aggregation progress of the client proteins was monitored by measuring light scattering at 360 nm as a function of time, using a T90^+^ UV–Vis spectrophotometer (PG Instrument Ltd., UK) equipped with a Peltier temperature controller. Moreover, all of the measurements were done in the absence of shaking/stirring condition.

Origin Pro 8.0 SR0 software was used for the calculations. To characterize the degree of agreement between experimental data and calculated values, we used the coefficient of determination *R*^2^ (see Ref. [6]).

## Funding

This work was supported by INSF (grant number 96008461), NIMAD (grant number 964854) and RSF (grant number 16-14-10055 to B.I.K.).
