# Supplementary material for: Kinetic data analysis of chaperone-like activity of Wt, R69C and D109H αB-crystallins
Source: Data Brief. 2019 Dec 4;28:104922. doi: 10.1016/j.dib.2019.104922 (PMC6939022; doi:10.1016/j.dib.2019.104922)
Supplement: Multimedia component 1 [file mmc1.docx]

**Supplementary data**

**Kinetic data analysis of chaperone-like activity of Wt, R69C and D109H αB-crystallins**

Maryam Ghahramani^1^, Reza Yousefi*^1^, Alexey Krivandin^2^, Konstantin Muranov^2^, Boris Kurganov^3^, Ali Akbar Moosavi-Movahedi^4^

^1^Protein Chemistry Laboratory (PCL), Department of Biology, College of Sciences, Shiraz University, Shiraz, Iran

^2^Emanuel Institute of Biochemical Physics, Russian Academy of Sciences, Kosygin str. 4, Moscow 119991, Russia.

^3^Bach Institute of Biochemistry, Research Center of Biotechnology of the Russian Academy of Sciences, 33, bld. 2 Leninsky Ave., Moscow 119071, Russia

^4^Institute of Biochemistry and Biophysics (IBB), University of Tehran, Tehran, Iran

* Corresponding author: Phone: +98 71 36137617, Fax: ++98 71 32280916.

E-mail: ryouseﬁ@shirazu.ac.ir

**Table S1. Kinetics of aggregation of insulin (0.3 mg·mL^-1^) in the presence of 20 mM DTT at 42 °C.** The dependences of the light scattering intensity at 360 nm on time for aggregation of insulin in the absence of any additives (column “Insulin”), in the presence of Wt αB-Cry (0.08 mg·mL^-1^; column “+ Wt”), in the presence of R69C mutant form of αB-Cry (0.08 mg·mL^-1^; column “+ R69C”) and in the presence of D109H mutant form of αB-Cry (0.08 mg·mL^-1^; column “+ D109H”).

| Time, min | Insulin | + Wt | + R69C | + D109H |
| --- | --- | --- | --- | --- |
| 0 | 0.007 | 0 | 0 | 0.0073 |
| 0.2 | 0.008 | 0 | 0 | 0.0087 |
| 0.4 | 0.008 | 0 | 0.0007 | 0.0090 |
| 0.6 | 0.009 | 0.003 | 0.0013 | 0.0107 |
| 0.8 | 0.013 | 0.005 | 0.0023 | 0.0120 |
| 1 | 0.018 | 0.008 | 0.0030 | 0.0140 |
| 1.2 | 0.027 | 0.0105 | 0.0057 | 0.0160 |
| 1.4 | 0.04 | 0.0105 | 0.0090 | 0.0190 |
| 1.6 | 0.06 | 0.0115 | 0.0137 | 0.0217 |
| 1.8 | 0.089 | 0.014 | 0.0197 | 0.0247 |
| 2 | 0.122 | 0.017 | 0.0260 | 0.0287 |
| 2.2 | 0.152 | 0.02 | 0.0333 | 0.0323 |
| 2.4 | 0.181 | 0.023 | 0.0400 | 0.0367 |
| 2.6 | 0.207 | 0.0255 | 0.0463 | 0.0407 |
| 2.8 | 0.231 | 0.028 | 0.0513 | 0.0447 |
| 3 | 0.252 | 0.0305 | 0.0580 | 0.0483 |
| 3.2 | 0.271 | 0.032 | 0.0633 | 0.0530 |
| 3.4 | 0.288 | 0.035 | 0.0677 | 0.0567 |
| 3.6 | 0.303 | 0.036 | 0.0717 | 0.0603 |
| 3.8 | 0.317 | 0.0375 | 0.0753 | 0.0647 |
| 4 | 0.33 | 0.0385 | 0.0783 | 0.0687 |
| 4.2 | 0.342 | 0.04 | 0.0813 | 0.0723 |
| 4.4 | 0.353 | 0.041 | 0.0837 | 0.0763 |
| 4.6 | 0.363 | 0.042 | 0.0857 | 0.0800 |
| 4.8 | 0.373 | 0.0435 | 0.0877 | 0.0840 |
| 5 | 0.382 | 0.044 | 0.0890 | 0.0880 |
| 5.2 | 0.391 | 0.0435 | 0.0903 | 0.0917 |
| 5.4 | 0.398 | 0.0445 | 0.0917 | 0.0957 |
| 5.6 | 0.406 | 0.045 | 0.0930 | 0.0987 |
| 5.8 | 0.413 | 0.0455 | 0.0940 | 0.1020 |
| 6 | 0.42 | 0.0465 | 0.0957 | 0.1060 |
| 6.2 | 0.427 | 0.0475 | 0.0973 | 0.1090 |
| 6.4 | 0.432 | 0.0475 | 0.0980 | 0.1123 |
| 6.6 | 0.438 | 0.0485 | 0.0997 | 0.1153 |
| 6.8 | 0.445 | 0.049 | 0.1007 | 0.1187 |
| 7 | 0.45 | 0.0495 | 0.1017 | 0.1220 |
| 7.2 | 0.455 | 0.0505 | 0.1030 | 0.1250 |
| 7.4 | 0.46 | 0.051 | 0.1037 | 0.1277 |
| 7.6 | 0.465 | 0.0515 | 0.1047 | 0.1300 |
| 7.8 | 0.469 | 0.0525 | 0.1057 | 0.1330 |
| 8 | 0.474 | 0.0535 | 0.1067 | 0.1353 |
| 8.2 | 0.479 | 0.054 | 0.1077 | 0.1387 |
| 8.4 | 0.483 | 0.054 | 0.1083 | 0.1410 |
| 8.6 | 0.487 | 0.0545 | 0.1093 | 0.1440 |
| 8.8 | 0.491 | 0.055 | 0.1103 | 0.1467 |
| 9 | 0.495 | 0.056 | 0.1113 | 0.1493 |
| 9.2 | 0.499 | 0.056 | 0.1123 | 0.1520 |
| 9.4 | 0.502 | 0.0565 | 0.1130 | 0.1530 |
| 9.6 | 0.505 | 0.0565 | 0.1137 | 0.1543 |
| 9.8 | 0.51 | 0.057 | 0.1147 | 0.1547 |
| 10 | 0.513 | 0.057 | 0.1153 | 0.1563 |
| 10.2 | 0.516 | 0.058 | 0.1157 | 0.1597 |
| 10.4 | 0.519 | 0.0575 | 0.1170 | 0.1637 |
| 10.6 | 0.523 | 0.0585 | 0.1177 | 0.1670 |
| 10.8 | 0.526 | 0.0585 | 0.1183 | 0.1700 |
| 11 | 0.529 | 0.0595 | 0.1190 | 0.1727 |
| 11.2 | 0.532 | 0.0595 | 0.1197 | 0.1753 |
| 11.4 | 0.535 | 0.06 | 0.1203 | 0.1780 |
| 11.6 | 0.537 | 0.0605 | 0.1203 | 0.1807 |
| 11.8 | 0.54 | 0.0615 | 0.1213 | 0.1837 |
| 12 | 0.542 | 0.0615 | 0.1213 | 0.1857 |
| 12.2 | 0.544 | 0.062 | 0.1220 | 0.1887 |
| 12.4 | 0.545 | 0.062 | 0.1227 | 0.1900 |
| 12.6 | 0.547 | 0.0625 | 0.1237 | 0.1927 |
| 12.8 | 0.548 | 0.0625 | 0.1237 | 0.1940 |
| 13 | 0.55 | 0.063 | 0.1243 | 0.1953 |
| 13.2 | 0.553 | 0.063 | 0.1247 | 0.1973 |
| 13.4 | 0.553 | 0.063 | 0.1257 | 0.1993 |
| 13.6 | 0.554 | 0.0635 | 0.1260 | 0.2013 |
| 13.8 | 0.555 | 0.0635 | 0.1263 | 0.2033 |
| 14 | 0.555 | 0.0645 | 0.1270 | 0.2047 |
| 14.2 | 0.558 | 0.0655 | 0.1277 | 0.2067 |
| 14.4 | 0.56 | 0.0655 | 0.1280 | 0.2080 |
| 14.6 | 0.561 | 0.066 | 0.1283 | 0.2093 |
| 14.8 | 0.563 | 0.0665 | 0.1290 | 0.2107 |
| 15 | 0.565 | 0.067 | 0.1297 | 0.2127 |
| 15.2 | 0.567 | 0.067 | 0.1303 | 0.2147 |
| 15.4 | 0.569 | 0.068 | 0.1310 | 0.2163 |
| 15.6 | 0.571 | 0.0685 | 0.1313 | 0.2180 |
| 15.8 | 0.573 | 0.0685 | 0.1313 | 0.2197 |
| 16 | 0.575 | 0.0695 | 0.1320 | 0.2210 |
| 16.2 | 0.577 | 0.07 | 0.1323 | 0.2217 |
| 16.4 | 0.58 | 0.07 | 0.1330 | 0.2210 |
| 16.6 | 0.582 | 0.0705 | 0.1330 | 0.2190 |
| 16.8 | 0.584 | 0.071 | 0.1333 | 0.2167 |
| 17 | 0.586 | 0.0715 | 0.1340 | 0.2170 |
| 17.2 | 0.588 | 0.0715 | 0.1343 | 0.2180 |
| 17.4 | 0.589 | 0.0725 | 0.1350 | 0.2190 |
| 17.6 | 0.592 | 0.073 | 0.1350 | 0.2217 |
| 17.8 | 0.593 | 0.073 | 0.1353 | 0.2240 |
| 18 | 0.595 | 0.074 | 0.1360 | 0.2263 |
| 18.2 | 0.596 | 0.0745 | 0.1363 | 0.2280 |
| 18.4 | 0.598 | 0.0745 | 0.1370 | 0.2293 |
| 18.6 | 0.6 | 0.0755 | 0.1370 | 0.2307 |
| 18.8 | 0.601 | 0.0755 | 0.1377 | 0.2320 |
| 19 | 0.603 | 0.076 | 0.1383 | 0.2340 |
| 19.2 | 0.604 | 0.077 | 0.1387 | 0.2360 |
| 19.4 | 0.607 | 0.0775 | 0.1397 | 0.2383 |
| 19.6 | 0.607 | 0.0775 | 0.1393 | 0.2410 |
| 19.8 | 0.609 | 0.078 | 0.1403 | 0.2427 |
| 20 | 0.611 | 0.079 | 0.1407 | 0.2443 |
| 20.2 | 0.612 | 0.0795 | 0.1410 | 0.2457 |
| 20.4 | 0.614 | 0.08 | 0.1420 | 0.2467 |
| 20.6 | 0.616 | 0.0805 | 0.1423 | 0.2480 |
| 20.8 | 0.617 | 0.0805 | 0.1430 | 0.2490 |
| 21 | 0.619 | 0.0815 | 0.1433 | 0.2503 |
| 21.2 | 0.621 | 0.0815 | 0.1490 | 0.2500 |
| 21.4 | 0.623 | 0.082 | 0.1510 | 0.2500 |
| 21.6 | 0.624 | 0.083 | 0.1515 | 0.2510 |
| 21.8 | 0.626 | 0.0835 | 0.1525 | 0.2520 |
| 22 | 0.627 | 0.084 | 0.1540 | 0.2520 |
| 22.2 | 0.629 | 0.0845 | 0.1545 | 0.2530 |
| 22.4 | 0.631 | 0.0855 | 0.1520 | 0.2540 |
| 22.6 | 0.632 | 0.085 | 0.1530 | 0.2540 |
| 22.8 | 0.634 | 0.0865 | 0.1530 | 0.2540 |
| 23 | 0.636 | 0.0865 | 0.1540 | 0.2550 |
| 23.2 | 0.638 | 0.087 | 0.1540 | 0.2560 |
| 23.4 | 0.638 | 0.088 | 0.1550 | 0.2570 |
| 23.6 | 0.64 | 0.0885 | 0.1550 | 0.2580 |
| 23.8 | 0.641 | 0.0885 | 0.1560 | 0.2590 |
| 24 | 0.644 | 0.0895 | 0.1560 | 0.2590 |
| 24.2 | 0.646 | 0.0895 | 0.1560 | 0.2610 |
| 24.4 | 0.649 | 0.09 | 0.1560 | 0.2610 |
| 24.6 | 0.651 | 0.09 | 0.1560 | 0.2620 |
| 24.8 | 0.652 | 0.0905 | 0.1570 | 0.2630 |
| 25 | 0.654 | 0.0915 | 0.1570 | 0.2630 |
| 25.2 | 0.657 | 0.0915 | 0.1570 | 0.2630 |
| 25.4 | 0.659 | 0.092 | 0.1570 | 0.2630 |
| 25.6 | 0.66 | 0.092 | 0.1570 | 0.2640 |
| 25.8 | 0.661 | 0.092 | 0.1580 | 0.2640 |
| 26 | 0.662 | 0.0935 | 0.1580 | 0.2650 |
| 26.2 | 0.663 | 0.0935 | 0.1580 | 0.2650 |
| 26.4 | 0.664 | 0.094 | 0.1580 | 0.2650 |
| 26.6 | 0.665 | 0.094 | 0.1580 | 0.2660 |
| 26.8 | 0.668 | 0.0945 | 0.1590 | 0.2660 |
| 27 | 0.669 | 0.0945 | 0.1590 | 0.2670 |
| 27.2 | 0.671 | 0.0955 | 0.1590 | 0.2670 |
| 27.4 | 0.672 | 0.096 | 0.1590 | 0.2670 |
| 27.6 | 0.674 | 0.096 | 0.1590 | 0.2670 |
| 27.8 | 0.674 | 0.096 | 0.1600 | 0.2660 |
| 28 | 0.675 | 0.0965 | 0.1600 | 0.2660 |
| 28.2 | 0.676 | 0.0975 | 0.1600 | 0.2650 |
| 28.4 | 0.677 | 0.0975 | 0.1600 | 0.2670 |
| 28.6 | 0.68 | 0.098 | 0.1600 | 0.2690 |
| 28.8 | 0.683 | 0.098 | 0.1600 | 0.2690 |
| 29 | 0.684 | 0.098 | 0.1620 | 0.2690 |
| 29.2 | 0.683 | 0.0995 | 0.1620 | 0.2700 |
| 29.4 | 0.685 | 0.0995 | 0.1620 | 0.2700 |
| 29.6 | 0.685 | 0.0995 | 0.1620 | 0.2700 |
| 29.8 | 0.688 | 0.1 | 0.1620 | 0.2700 |
| 30 | 0.69 | 0.1 | 0.1620 | 0.2700 |

**Table S2. Kinetics of aggregation of catalase (0.3 mg·mL^-1^) at 60 °C.** The dependences of the light scattering intensity at 360 nm on time for aggregation of insulin in the absence of any additives (column “Catalase”), in the presence of Wt αB-Cry (0.08 mg·mL^-1^; column “+ Wt”), in the presence of R69C mutant form of αB-Cry (0.08 mg·mL^-1^; column “+ R69C”) and in the presence of D109H mutant form of αB-Cry (0.08 mg·mL^-1^; column “+ D109H”).

| Time, min | Catalase | + Wt | + R69C | + D109H |
| --- | --- | --- | --- | --- |
| 0 | 0.001 | 0 | -0.001 | 0.0003 |
| 0.2 | 0.002 | 0 | -0.003 | 0.0020 |
| 0.4 | 0.01 | 0.014 | -0.003 | -0.0023 |
| 0.6 | 0.001 | 0.005 | -0.003 | -0.0027 |
| 0.8 | 0.003 | 0 | -0.004 | -0.0043 |
| 1 | 0.002 | 0.005 | -0.003 | -0.0043 |
| 1.2 | 0.001 | 0.004 | -0.003 | -0.0047 |
| 1.4 | 0.001 | 0.003 | -0.002 | -0.0033 |
| 1.6 | 0.003 | 0.004 | 0 | -0.0007 |
| 1.8 | 0.01 | 0.008 | 0.004 | 0.0037 |
| 2 | 0.023 | 0.009 | 0.006 | 0.0070 |
| 2.2 | 0.04 | 0.011 | 0.008 | 0.0143 |
| 2.4 | 0.064 | 0.016 | 0.01 | 0.0227 |
| 2.6 | 0.093 | 0.019 | 0.014 | 0.0350 |
| 2.8 | 0.128 | 0.021 | 0.015 | 0.0477 |
| 3 | 0.167 | 0.025 | 0.017 | 0.0640 |
| 3.2 | 0.209 | 0.028 | 0.018 | 0.0823 |
| 3.4 | 0.249 | 0.033 | 0.021 | 0.1013 |
| 3.6 | 0.287 | 0.037 | 0.021 | 0.1207 |
| 3.8 | 0.323 | 0.038 | 0.021 | 0.1407 |
| 4 | 0.355 | 0.041 | 0.022 | 0.1613 |
| 4.2 | 0.384 | 0.044 | 0.023 | 0.1807 |
| 4.4 | 0.41 | 0.046 | 0.024 | 0.2000 |
| 4.6 | 0.434 | 0.048 | 0.025 | 0.2183 |
| 4.8 | 0.454 | 0.051 | 0.026 | 0.2360 |
| 5 | 0.474 | 0.053 | 0.026 | 0.2533 |
| 5.2 | 0.49 | 0.053 | 0.026 | 0.2687 |
| 5.4 | 0.506 | 0.056 | 0.027 | 0.2830 |
| 5.6 | 0.518 | 0.056 | 0.027 | 0.2977 |
| 5.8 | 0.532 | 0.058 | 0.028 | 0.3107 |
| 6 | 0.543 | 0.059 | 0.028 | 0.3227 |
| 6.2 | 0.553 | 0.061 | 0.028 | 0.3340 |
| 6.4 | 0.563 | 0.062 | 0.029 | 0.3447 |
| 6.6 | 0.572 | 0.062 | 0.03 | 0.3560 |
| 6.8 | 0.58 | 0.064 | 0.03 | 0.3660 |
| 7 | 0.588 | 0.065 | 0.03 | 0.3757 |
| 7.2 | 0.596 | 0.065 | 0.03 | 0.3847 |
| 7.4 | 0.602 | 0.066 | 0.03 | 0.3940 |
| 7.6 | 0.609 | 0.068 | 0.03 | 0.4023 |
| 7.8 | 0.614 | 0.068 | 0.03 | 0.4110 |
| 8 | 0.62 | 0.07 | 0.03 | 0.4190 |
| 8.2 | 0.625 | 0.07 | 0.031 | 0.4267 |
| 8.4 | 0.63 | 0.071 | 0.031 | 0.4340 |
| 8.6 | 0.636 | 0.072 | 0.031 | 0.4403 |
| 8.8 | 0.64 | 0.073 | 0.031 | 0.4470 |
| 9 | 0.645 | 0.074 | 0.031 | 0.4527 |
| 9.2 | 0.649 | 0.075 | 0.031 | 0.4587 |
| 9.4 | 0.653 | 0.074 | 0.031 | 0.4637 |
| 9.6 | 0.658 | 0.076 | 0.031 | 0.4690 |
| 9.8 | 0.662 | 0.076 | 0.032 | 0.4737 |
| 10 | 0.666 | 0.077 | 0.032 | 0.4787 |
| 10.2 | 0.67 | 0.079 | 0.032 | 0.4840 |
| 10.4 | 0.673 | 0.078 | 0.032 | 0.4880 |
| 10.6 | 0.677 | 0.08 | 0.032 | 0.4920 |
| 10.8 | 0.681 | 0.079 | 0.032 | 0.4967 |
| 11 | 0.685 | 0.08 | 0.033 | 0.5007 |
| 11.2 | 0.688 | 0.081 | 0.032 | 0.5047 |
| 11.4 | 0.693 | 0.082 | 0.033 | 0.5083 |
| 11.6 | 0.696 | 0.081 | 0.032 | 0.5117 |
| 11.8 | 0.698 | 0.081 | 0.032 | 0.5153 |
| 12 | 0.703 | 0.081 | 0.032 | 0.5190 |
| 12.2 | 0.706 | 0.083 | 0.033 | 0.5213 |
| 12.4 | 0.709 | 0.083 | 0.033 | 0.5247 |
| 12.6 | 0.711 | 0.082 | 0.033 | 0.5270 |
| 12.8 | 0.715 | 0.084 | 0.033 | 0.5300 |
| 13 | 0.718 | 0.085 | 0.033 | 0.5320 |
| 13.2 | 0.72 | 0.084 | 0.033 | 0.5347 |
| 13.4 | 0.723 | 0.085 | 0.033 | 0.5370 |
| 13.6 | 0.725 | 0.085 | 0.033 | 0.5390 |
| 13.8 | 0.728 | 0.085 | 0.033 | 0.5413 |
| 14 | 0.73 | 0.085 | 0.033 | 0.5433 |
| 14.2 | 0.734 | 0.086 | 0.033 | 0.5457 |
| 14.4 | 0.736 | 0.086 | 0.033 | 0.5480 |
| 14.6 | 0.739 | 0.086 | 0.033 | 0.5497 |
| 14.8 | 0.74 | 0.086 | 0.033 | 0.5510 |
| 15 | 0.743 | 0.088 | 0.033 | 0.5527 |
| 15.2 | 0.745 | 0.087 | 0.033 | 0.5547 |
| 15.4 | 0.747 | 0.087 | 0.033 | 0.5557 |
| 15.6 | 0.749 | 0.088 | 0.033 | 0.5573 |
| 15.8 | 0.751 | 0.089 | 0.033 | 0.5580 |
| 16 | 0.754 | 0.088 | 0.033 | 0.5600 |
| 16.2 | 0.755 | 0.089 | 0.033 | 0.5610 |
| 16.4 | 0.757 | 0.089 | 0.034 | 0.5627 |
| 16.6 | 0.759 | 0.089 | 0.034 | 0.5637 |
| 16.8 | 0.761 | 0.09 | 0.034 | 0.5647 |
| 17 | 0.763 | 0.09 | 0.033 | 0.5653 |
| 17.2 | 0.765 | 0.09 | 0.034 | 0.5657 |
| 17.4 | 0.767 | 0.091 | 0.034 | 0.5673 |
| 17.6 | 0.769 | 0.091 | 0.034 | 0.5683 |
| 17.8 | 0.77 | 0.092 | 0.034 | 0.5707 |
| 18 | 0.772 | 0.092 | 0.034 | 0.5713 |
| 18.2 | 0.774 | 0.092 | 0.034 | 0.5730 |
| 18.4 | 0.776 | 0.092 | 0.034 | 0.5737 |
| 18.6 | 0.778 | 0.092 | 0.034 | 0.5753 |
| 18.8 | 0.78 | 0.092 | 0.034 | 0.5767 |
| 19 | 0.781 | 0.092 | 0.034 | 0.5790 |
| 19.2 | 0.783 | 0.093 | 0.034 | 0.5793 |
| 19.4 | 0.785 | 0.093 | 0.034 | 0.5800 |
| 19.6 | 0.787 | 0.092 | 0.034 | 0.5810 |
| 19.8 | 0.789 | 0.093 | 0.034 | 0.5813 |
| 20 | 0.791 | 0.093 | 0.034 | 0.5833 |
| 20.2 | 0.793 | 0.093 | 0.034 | 0.5843 |
| 20.4 | 0.794 | 0.093 | 0.035 | 0.5843 |
| 20.6 | 0.794 | 0.093 | 0.035 | 0.5850 |
| 20.8 | 0.795 | 0.094 | 0.035 | 0.5857 |
| 21 | 0.796 | 0.094 | 0.035 | 0.5857 |
| 21.2 | 0.798 | 0.094 | 0.035 | 0.5867 |
| 21.4 | 0.799 | 0.094 | 0.035 | 0.5867 |
| 21.6 | 0.8 | 0.094 | 0.035 | 0.5877 |
| 21.8 | 0.801 | 0.094 | 0.035 | 0.5877 |
| 22 | 0.801 | 0.094 | 0.035 | 0.5880 |
| 22.2 | 0.803 | 0.094 | 0.035 | 0.5883 |
| 22.4 | 0.803 | 0.095 | 0.035 | 0.5887 |
| 22.6 | 0.805 | 0.094 | 0.035 | 0.5883 |
| 22.8 | 0.806 | 0.095 | 0.035 | 0.5880 |
| 23 | 0.808 | 0.095 | 0.035 | 0.5883 |
| 23.2 | 0.809 | 0.096 | 0.035 | 0.5887 |
| 23.4 | 0.809 | 0.095 | 0.035 | 0.5897 |
| 23.6 | 0.812 | 0.096 | 0.035 | 0.5893 |
| 23.8 | 0.812 | 0.096 | 0.035 | 0.5903 |
| 24 | 0.814 | 0.095 | 0.035 | 0.5897 |
| 24.2 | 0.814 | 0.096 | 0.035 | 0.5900 |
| 24.4 | 0.816 | 0.096 | 0.035 | 0.5903 |
| 24.6 | 0.816 | 0.096 | 0.036 | 0.5910 |
| 24.8 | 0.818 | 0.097 | 0.036 | 0.5907 |
| 25 | 0.819 | 0.097 | 0.036 | 0.5910 |
| 25.2 | 0.82 | 0.097 | 0.036 | 0.5917 |
| 25.4 | 0.821 | 0.096 | 0.036 | 0.5917 |
| 25.6 | 0.822 | 0.096 | 0.036 | 0.5907 |
| 25.8 | 0.825 | 0.097 | 0.036 | 0.5900 |
| 26 | 0.825 | 0.097 | 0.036 | 0.5903 |
| 26.2 | 0.826 | 0.097 | 0.036 | 0.5923 |
| 26.4 | 0.828 | 0.097 | 0.036 | 0.5927 |
| 26.6 | 0.828 | 0.097 | 0.036 | 0.5920 |
| 26.8 | 0.83 | 0.098 | 0.036 | 0.5903 |
| 27 | 0.831 | 0.098 | 0.036 | 0.5900 |
| 27.2 | 0.829 | 0.098 | 0.036 | 0.5913 |
| 27.4 | 0.83 | 0.097 | 0.036 | 0.5930 |
| 27.6 | 0.831 | 0.097 | 0.036 | 0.5940 |
| 27.8 | 0.832 | 0.098 | 0.037 | 0.5933 |
| 28 | 0.833 | 0.098 | 0.037 | 0.5920 |
| 28.2 | 0.833 | 0.098 | 0.037 | 0.5927 |
| 28.4 | 0.832 | 0.098 | 0.037 | 0.5937 |
| 28.6 | 0.833 | 0.099 | 0.037 | 0.5950 |
| 28.8 | 0.833 | 0.098 | 0.037 | 0.5963 |
| 29 | 0.836 | 0.099 | 0.037 | 0.5947 |
| 29.2 | 0.834 | 0.098 | 0.037 | 0.5930 |
| 29.4 | 0.837 | 0.098 | 0.037 | 0.5930 |
| 29.6 | 0.837 | 0.098 | 0.037 | 0.5947 |
| 29.8 | 0.837 | 0.098 | 0.037 | 0.5963 |
| 30 | 0.837 | 0.099 | 0.037 | 0.5960 |

**Table S3. Kinetics of aggregation of lysozyme (0.2 mg·mL^-1^) in the presence of 20 mM DTT at 42 °C.** The dependences of the light scattering intensity at 360 nm on time for aggregation of lysozyme in the absence of any additives (column “Lysozyme”), in the presence of Wt αB-Cry (0.08 mg·mL^-1^; column “+ Wt”), in the presence of R69C mutant form of αB-Cry (0.08 mg·mL^-1^; column “+ R69C”) and in the presence of D109H mutant form of αB-Cry (0.08 mg·mL^-1^; column “+ D109H”).

| Time, min | Lysozyme | + Wt | + R69C | + D109H |
| --- | --- | --- | --- | --- |
| 0 | 0.001 | 0.0005 | 0.0005 | 0.001 |
| 0.5 | 0.002 | 0.0015 | 0.0005 | 0.0025 |
| 1 | 0.009 | -0.0005 | -0.001 | 0.001 |
| 1.5 | 0.016 | 0.0025 | -0.001 | 0.0015 |
| 2 | 0.022 | -0.0005 | -0.0005 | 0.001 |
| 2.5 | 0.028 | -0.0005 | -0.001 | 0.002 |
| 3 | 0.033 | 0.0005 | 0 | 0.002 |
| 3.5 | 0.038 | 0.0015 | 0.0005 | 0.003 |
| 4 | 0.043 | 0.0015 | 0.001 | 0.0035 |
| 4.5 | 0.048 | 0.0025 | 0.0025 | 0.0045 |
| 5 | 0.054 | 0.0035 | 0.003 | 0.005 |
| 5.5 | 0.06 | 0.0035 | 0.004 | 0.0055 |
| 6 | 0.068 | 0.0045 | 0.005 | 0.0065 |
| 6.5 | 0.077 | 0.0045 | 0.0055 | 0.007 |
| 7 | 0.087 | 0.0055 | 0.007 | 0.0075 |
| 7.5 | 0.099 | 0.0065 | 0.008 | 0.0085 |
| 8 | 0.115 | 0.0075 | 0.0085 | 0.009 |
| 8.5 | 0.133 | 0.008 | 0.01 | 0.01 |
| 9 | 0.153 | 0.0085 | 0.0115 | 0.011 |
| 9.5 | 0.177 | 0.0095 | 0.013 | 0.0115 |
| 10 | 0.203 | 0.01 | 0.0145 | 0.013 |
| 10.5 | 0.232 | 0.011 | 0.0165 | 0.0145 |
| 11 | 0.264 | 0.0115 | 0.019 | 0.0155 |
| 11.5 | 0.298 | 0.0125 | 0.021 | 0.0165 |
| 12 | 0.332 | 0.013 | 0.024 | 0.018 |
| 12.5 | 0.367 | 0.0145 | 0.0265 | 0.02 |
| 13 | 0.403 | 0.015 | 0.03 | 0.0215 |
| 13.5 | 0.438 | 0.0165 | 0.033 | 0.023 |
| 14 | 0.473 | 0.0175 | 0.0375 | 0.0255 |
| 14.5 | 0.506 | 0.018 | 0.042 | 0.0275 |
| 15 | 0.536 | 0.0195 | 0.0465 | 0.03 |
| 15.5 | 0.565 | 0.021 | 0.052 | 0.0325 |
| 16 | 0.594 | 0.022 | 0.0585 | 0.036 |
| 16.5 | 0.62 | 0.0235 | 0.0645 | 0.039 |
| 17 | 0.645 | 0.0245 | 0.072 | 0.043 |
| 17.5 | 0.669 | 0.0265 | 0.081 | 0.047 |
| 18 | 0.693 | 0.028 | 0.09 | 0.052 |
| 18.5 | 0.715 | 0.0305 | 0.1 | 0.058 |
| 19 | 0.737 | 0.033 | 0.1105 | 0.0645 |
| 19.5 | 0.757 | 0.0345 | 0.121 | 0.071 |
| 20 | 0.777 | 0.0375 | 0.133 | 0.08 |
| 20.5 | 0.795 | 0.04 | 0.146 | 0.0895 |
| 21 | 0.813 | 0.0435 | 0.16 | 0.1 |
| 21.5 | 0.828 | 0.0465 | 0.1735 | 0.1115 |
| 22 | 0.841 | 0.05 | 0.1885 | 0.124 |
| 22.5 | 0.856 | 0.054 | 0.203 | 0.1385 |
| 23 | 0.871 | 0.058 | 0.218 | 0.1535 |
| 23.5 | 0.882 | 0.063 | 0.234 | 0.1705 |
| 24 | 0.896 | 0.0685 | 0.2495 | 0.1885 |
| 24.5 | 0.908 | 0.0745 | 0.267 | 0.2085 |
| 25 | 0.92 | 0.081 | 0.284 | 0.23 |
| 25.5 | 0.93 | 0.0885 | 0.301 | 0.2535 |
| 26 | 0.94 | 0.0965 | 0.3175 | 0.279 |
| 26.5 | 0.948 | 0.1045 | 0.3335 | 0.3055 |
| 27 | 0.956 | 0.1145 | 0.3485 | 0.3335 |
| 27.5 | 0.965 | 0.1245 | 0.365 | 0.36 |
| 28 | 0.973 | 0.136 | 0.3815 | 0.388 |
| 28.5 | 0.982 | 0.148 | 0.3975 | 0.4145 |
| 29 | 0.987 | 0.161 | 0.4135 | 0.4405 |
| 29.5 | 0.993 | 0.176 | 0.4285 | 0.465 |
| 30 | 0.999 | 0.1915 | 0.442 | 0.49 |
| 30.5 | 1.005 | 0.2085 | 0.457 | 0.513 |
| 31 | 1.012 | 0.226 | 0.4715 | 0.5365 |
| 31.5 | 1.018 | 0.2435 | 0.486 | 0.5575 |
| 32 | 1.024 | 0.262 | 0.5 | 0.5785 |
| 32.5 | 1.032 | 0.2815 | 0.512 | 0.5975 |
| 33 | 1.037 | 0.3025 | 0.524 | 0.6145 |
| 33.5 | 1.042 | 0.324 | 0.5365 | 0.6295 |
| 34 | 1.047 | 0.346 | 0.55 | 0.647 |
| 34.5 | 1.049 | 0.3695 | 0.5625 | 0.663 |
| 35 | 1.055 | 0.39 | 0.5745 | 0.6775 |
| 35.5 | 1.06 | 0.4095 | 0.587 | 0.6895 |
| 36 | 1.069 | 0.4285 | 0.598 | 0.7015 |
| 36.5 | 1.075 | 0.448 | 0.608 | 0.714 |
| 37 | 1.081 | 0.4685 | 0.619 | 0.725 |
| 37.5 | 1.086 | 0.49 | 0.63 | 0.737 |
| 38 | 1.087 | 0.51 | 0.6395 | 0.7475 |
| 38.5 | 1.09 | 0.527 | 0.6495 | 0.7585 |
| 39 | 1.091 | 0.5415 | 0.659 | 0.7675 |
| 39.5 | 1.092 | 0.5545 | 0.6675 | 0.778 |
| 40 | 1.096 | 0.5685 | 0.676 | 0.7865 |
| 40.5 | 1.102 | 0.585 | 0.6875 | 0.796 |
| 41 | 1.109 | 0.6005 | 0.695 | 0.805 |
| 41.5 | 1.119 | 0.6125 | 0.7055 | 0.813 |
| 42 | 1.123 | 0.6195 | 0.7145 | 0.8215 |
| 42.5 | 1.126 | 0.6245 | 0.7245 | 0.8295 |
| 43 | 1.127 | 0.629 | 0.736 | 0.837 |
| 43.5 | 1.127 | 0.6365 | 0.7435 | 0.845 |
| 44 | 1.127 | 0.6475 | 0.7495 | 0.852 |
| 44.5 | 1.129 | 0.659 | 0.756 | 0.858 |
| 45 | 1.133 | 0.6685 | 0.7615 | 0.864 |
| 45.5 | 1.138 | 0.67 | 0.7665 | 0.87 |
| 46 | 1.144 | 0.68 | 0.7725 | 0.877 |
| 46.5 | 1.148 | 0.689 | 0.7775 | 0.8825 |
| 47 | 1.152 | 0.695 | 0.7845 | 0.8895 |
| 47.5 | 1.152 | 0.701 | 0.79 | 0.8975 |
| 48 | 1.152 | 0.71 | 0.794 | 0.904 |
| 48.5 | 1.149 | 0.715 | 0.7975 | 0.911 |
| 49 | 1.145 | 0.718 | 0.802 | 0.914 |
| 49.5 | 1.15 | 0.72 | 0.8055 | 0.9175 |
| 50 | 1.158 | 0.7135 | 0.81 | 0.9215 |
| 50.5 | 1.164 | 0.714 | 0.814 | 0.9256 |
| 51 | 1.167 | 0.716 | 0.818 | 0.928 |
| 51.5 | 1.164 | 0.719 | 0.822 | 0.931 |
| 52 | 1.161 | 0.72 | 0.825 | 0.9346 |
| 52.5 | 1.161 | 0.723 | 0.827 | 0.936 |
| 53 | 1.162 | 0.726 | 0.828 | 0.9389 |
| 53.5 | 1.165 | 0.727 | 0.829 | 0.942 |
| 54 | 1.171 | 0.729 | 0.831 | 0.945 |
| 54.5 | 1.185 | 0.73 | 0.833 | 0.948 |
| 55 | 1.193 | 0.735 | 0.835 | 0.949 |
| 55.5 | 1.19 | 0.738 | 0.837 | 0.9556 |
| 56 | 1.184 | 0.739 | 0.839 | 0.9578 |
| 56.5 | 1.178 | 0.742 | 0.84 | 0.9589 |
| 57 | 1.174 | 0.745 | 0.842 | 0.96 |
| 57.5 | 1.172 | 0.747 | 0.845 | 0.963 |
| 58 | 1.169 | 0.749 | 0.848 | 0.967 |
| 58.5 | 1.177 | 0.75 | 0.849 | 0.968 |
| 59 | 1.181 | 0.752 | 0.85 | 0.97 |
| 59.5 | 1.178 | 0.753 | 0.853 | 0.975 |
| 60 | 1.174 | 0.7553 | 0.854 | 0.978 |

**Table S4. Kinetics of aggregation of γ-Cry (0.16 mg·mL^-1^) at 60 °C.** The dependences of the light scattering intensity at 360 nm on time for aggregation of γ-Cry in the absence of any additives (column “γ-Cry”), in the presence of Wt αB-Cry (0.08 mg·mL^-1^; column “+ Wt”), in the presence of R69C mutant form of αB-Cry (0.08 mg·mL^-1^; column “+ R69C”) and in the presence of D109H mutant form of αB-Cry (0.08 mg·mL^-1^; column “+ D109H”).

| Time, min | γ-Cry | + Wt | + R69C | + D109H |
| --- | --- | --- | --- | --- |
| 0 | 0 | 0 | -0.0005 | 0 |
| 0.5 | 0 | 0.002 | -0.0005 | 0.001 |
| 1 | 0 | 0.001 | -0.001 | 0.001 |
| 1.5 | -0.001 | -0.001 | -0.001 | 0.001 |
| 2 | -0.001 | -0.001 | -0.002 | 0.0015 |
| 2.5 | -0.002 | -0.001 | -0.002 | 0.002 |
| 3 | -0.001 | -0.002 | -0.002 | 0.0025 |
| 3.5 | 0 | -0.002 | -0.0015 | 0.005 |
| 4 | -0.003 | -0.002 | -0.001 | 0.007 |
| 4.5 | -0.002 | -0.002 | -0.0005 | 0.01 |
| 5 | 0 | -0.003 | 0 | 0.014 |
| 5.5 | 0.005 | -0.002 | 0.001 | 0.018 |
| 6 | 0.012 | -0.001 | 0.001 | 0.0225 |
| 6.5 | 0.021 | -0.001 | 0.0015 | 0.028 |
| 7 | 0.032 | -0.001 | 0.0025 | 0.033 |
| 7.5 | 0.047 | -0.001 | 0.003 | 0.039 |
| 8 | 0.065 | 0.001 | 0.004 | 0.045 |
| 8.5 | 0.088 | 0.002 | 0.0045 | 0.05 |
| 9 | 0.112 | 0.002 | 0.006 | 0.0565 |
| 9.5 | 0.136 | 0.003 | 0.007 | 0.0615 |
| 10 | 0.161 | 0.003 | 0.008 | 0.0685 |
| 10.5 | 0.185 | 0.005 | 0.009 | 0.0745 |
| 11 | 0.209 | 0.007 | 0.0105 | 0.0815 |
| 11.5 | 0.233 | 0.009 | 0.011 | 0.089 |
| 12 | 0.261 | 0.011 | 0.0125 | 0.098 |
| 12.5 | 0.289 | 0.012 | 0.0145 | 0.1075 |
| 13 | 0.319 | 0.015 | 0.0165 | 0.117 |
| 13.5 | 0.347 | 0.018 | 0.0185 | 0.127 |
| 14 | 0.374 | 0.021 | 0.0215 | 0.1385 |
| 14.5 | 0.4 | 0.024 | 0.0245 | 0.1505 |
| 15 | 0.424 | 0.029 | 0.029 | 0.1645 |
| 15.5 | 0.451 | 0.033 | 0.033 | 0.1795 |
| 16 | 0.479 | 0.039 | 0.038 | 0.195 |
| 16.5 | 0.508 | 0.043 | 0.0435 | 0.211 |
| 17 | 0.535 | 0.049 | 0.0515 | 0.2295 |
| 17.5 | 0.562 | 0.055 | 0.058 | 0.2475 |
| 18 | 0.587 | 0.062 | 0.068 | 0.2685 |
| 18.5 | 0.608 | 0.069 | 0.0805 | 0.289 |
| 19 | 0.627 | 0.076 | 0.095 | 0.3125 |
| 19.5 | 0.652 | 0.084 | 0.109 | 0.3385 |
| 20 | 0.674 | 0.093 | 0.128 | 0.3635 |
| 20.5 | 0.698 | 0.103 | 0.1485 | 0.389 |
| 21 | 0.723 | 0.112 | 0.17 | 0.415 |
| 21.5 | 0.745 | 0.126 | 0.1965 | 0.44 |
| 22 | 0.764 | 0.137 | 0.2195 | 0.4635 |
| 22.5 | 0.781 | 0.15 | 0.2455 | 0.4865 |
| 23 | 0.796 | 0.165 | 0.2705 | 0.5095 |
| 23.5 | 0.811 | 0.177 | 0.2905 | 0.5305 |
| 24 | 0.83 | 0.192 | 0.323 | 0.551 |
| 24.5 | 0.851 | 0.209 | 0.3435 | 0.5695 |
| 25 | 0.87 | 0.221 | 0.3745 | 0.5865 |
| 25.5 | 0.889 | 0.239 | 0.411 | 0.6005 |
| 26 | 0.904 | 0.257 | 0.44 | 0.6135 |
| 26.5 | 0.917 | 0.273 | 0.4755 | 0.6265 |
| 27 | 0.929 | 0.291 | 0.5065 | 0.637 |
| 27.5 | 0.937 | 0.306 | 0.538 | 0.651 |
| 28 | 0.947 | 0.323 | 0.567 | 0.6585 |
| 28.5 | 0.961 | 0.343 | 0.588 | 0.669 |
| 29 | 0.974 | 0.362 | 0.6115 | 0.6775 |
| 29.5 | 0.986 | 0.372 | 0.6315 | 0.683 |
| 30 | 1 | 0.389 | 0.65 | 0.692 |
| 30.5 | 1.011 | 0.405 | 0.667 | 0.6975 |
| 31 | 1.019 | 0.423 | 0.6845 | 0.7035 |
| 31.5 | 1.023 | 0.435 | 0.6965 | 0.7095 |
| 32 | 1.03 | 0.449 | 0.7075 | 0.7165 |
| 32.5 | 1.037 | 0.468 | 0.717 | 0.719 |
| 33 | 1.046 | 0.481 | 0.726 | 0.7245 |
| 33.5 | 1.054 | 0.491 | 0.732 | 0.7285 |
| 34 | 1.063 | 0.505 | 0.74 | 0.733 |
| 34.5 | 1.071 | 0.517 | 0.7465 | 0.7365 |
| 35 | 1.077 | 0.529 | 0.7525 | 0.7465 |
| 35.5 | 1.078 | 0.541 | 0.7585 | 0.756 |
| 36 | 1.083 | 0.553 | 0.763 | 0.7545 |
| 36.5 | 1.085 | 0.562 | 0.769 | 0.7525 |
| 37 | 1.093 | 0.572 | 0.773 | 0.757 |
| 37.5 | 1.098 | 0.581 | 0.778 | 0.7515 |
| 38 | 1.106 | 0.591 | 0.781 | 0.754 |
| 38.5 | 1.108 | 0.601 | 0.783 | 0.753 |
| 39 | 1.109 | 0.609 | 0.784 | 0.7515 |
| 39.5 | 1.114 | 0.617 | 0.7875 | 0.7495 |
| 40 | 1.117 | 0.624 | 0.792 | 0.759 |
| 40.5 | 1.123 | 0.627 | 0.7945 | 0.759 |
| 41 | 1.127 | 0.633 | 0.797 | 0.756 |
| 41.5 | 1.127 | 0.638 | 0.798 | 0.758 |
| 42 | 1.127 | 0.645 | 0.8005 | 0.754 |
| 42.5 | 1.128 | 0.653 | 0.801 | 0.766 |
| 43 | 1.128 | 0.659 | 0.801 | 0.766 |
| 43.5 | 1.128 | 0.664 | 0.801 | 0.766 |
| 44 | 1.128 | 0.669 | 0.801 | 0.769 |
| 44.5 | 1.128 | 0.673 | 0.805 | 0.77 |
| 45 | 1.128 | 0.673 | 0.805 | 0.77 |
| 45.5 | 1.129 | 0.676 | 0.805 | 0.77 |
| 46 | 1.129 | 0.681 | 0.805 | 0.775 |
| 46.5 | 1.129 | 0.683 | 0.805 | 0.775 |
| 47 | 1.129 | 0.686 | 0.805 | 0.775 |
| 47.5 | 1.129 | 0.691 | 0.807 | 0.775 |
| 48 | 1.129 | 0.693 | 0.807 | 0.775 |
| 48.5 | 1.129 | 0.696 | 0.807 | 0.779 |
| 49 | 1.129 | 0.697 | 0.807 | 0.779 |
| 49.5 | 1.132 | 0.694 | 0.807 | 0.779 |
| 50 | 1.132 | 0.699 | 0.807 | 0.779 |
| 50.5 | 1.132 | 0.694 | 0.81 | 0.779 |
| 51 | 1.132 | 0.702 | 0.81 | 0.78 |
| 51.5 | 1.132 | 0.703 | 0.81 | 0.78 |
| 52 | 1.132 | 0.693 | 0.81 | 0.78 |
| 52.5 | 1.132 | 0.706 | 0.81 | 0.78 |
| 53 | 1.13 | 0.704 | 0.81 | 0.788 |
| 53.5 | 1.133 | 0.702 | 0.814 | 0.788 |
| 54 | 1.133 | 0.707 | 0.814 | 0.788 |
| 54.5 | 1.133 | 0.708 | 0.814 | 0.788 |
| 55 | 1.136 | 0.708 | 0.814 | 0.788 |
| 55.5 | 1.1326 | 0.715 | 0.814 | 0.79 |
| 56 | 1.136 | 0.715 | 0.814 | 0.79 |
| 56.5 | 1.136 | 0.724 | 0.816 | 0.79 |
| 57 | 1.138 | 0.721 | 0.816 | 0.79 |
| 57.5 | 1.138 | 0.723 | 0.816 | 0.79 |
| 58 | 1.138 | 0.723 | 0.816 | 0.794 |
| 58.5 | 1.14 | 0.723 | 0.816 | 0.794 |
| 59 | 1.14 | 0.722 | 0.819 | 0.794 |
| 59.5 | 1.141 | 0.722 | 0.819 | 0.794 |
| 60 | 1.141 | 0.722 | 0.819 | 0.794 |
